# Some Cases of Epistaxis and Other Hemorrhages

**Published:** 1880-01-20

**Authors:** Randolph Winslow

**Affiliations:** Assistant Demonstrator, University of Maryland


### *SOME CASES OF EPISTAXIS, AND OTHER HEMORRHAGES.*

BY RANDOLPH WINSLOW, M. D.,
Assistant Demonstrator, University of Maryland.

Epistaxis is one of those affections which every physician is called upon to treat. Generally it is an easy matter to arrest it, by cold applications to the face—ice held in the mouth—cold affusion or ice bag to the spine, etc., but, exceptionally, cases occur which require mechanical means to stop the hemorrhage and save life. The usual method adopted in such cases is to plug the nostrils by means of Belloc's canula, or a flexible catheter. When one has the necessary instruments, this is generally an easy and satisfactory way of accomplishing our purpose, but frequently a physician is called upon when he does not have these appliances, and it becomes necessary to resort to other means.

Having been accustomed for several years to arrest dangerous epistaxis by tamponing the nose with cotton, I desire to call attention to its advantages. Small pieces of cotton may easily be pushed along the nasal floor to any desired distance, or may be carried entirely through the nose from anterior nares to throat. The cotton should be saturated with water, and then with some astringent solution. If the cotton is not wet, the blood will slip by it without clotting. The liq. ferri persulph. is the most certain astringent for local use upon the cotton, but if that is not at hand we may use tr. ferri chlor., fl. ext. ergot, fl. ext. hamamelis, ol. terebinthinæ, solutions of alum, tannin or any other astringent which may be accessible. A female catheter, grooved director or almost any other long slender instrument may be used for pushing the cotton through the nose, or it may be applied upon a uterine applicator and slipped off in the nasal cavity by the wire spring surrounding the applicator. This method is inexpensive, and cotton can nearly always be obtained in a few minutes. It causes as little or less discomfort than the plug in the pharynx. It may be applied in every case; whilst in some instances it is difficult or impossible to plug the posterior nares from the mouth, owing to the reflex nausea and straining caused by the necessary manipulations. In some cases the difficulty of depressing the tongue prevents the use of the post nasal plug; especially is this apt to be the case with negroes.

The following four cases are copied from my note book, to show the results of the treatment in some severe cases in which I have employed it, and to call attention to the variety of astringents used. As far as I am aware, no injury has been done to the nasal structures in these or in any other cases which I have treated in this manner.

In August, 1876, I was called to attend E. S., aged about 40, who was having severe epistaxis. She had been bleeding more or less for a week, and had lost immense quantities of blood. She had received the attention of three regular and two homœopathic physicians, but beyond a very temporary arrest of the hemorrhage, she had not been benefited. Amongst other measures she had been tamponed by means of Belloc's canula, but without success, and probably the plug did not fit the nasal openings accurately. I saturated small pieces of cotton with liq. ferri persulph. and pushed them along the nasal floor, as far as was necessary, and thus filled the nose. Her after treatment consisted of 2 grains acetate lead and one-half grain opium every two hours, until 48 grains of lead had been taken, when she began to exhibit the toxic effects of the mineral; a blue line along the edge of the gums, and severe abdominal pain. Gallic acid was then substituted. Ergot was not used, as she was in the eighth month of pregnancy. Whilst scarcely able to walk, on account of prostration from loss of blood, her husband, who had previously said he would give $100 to have her cured, knocked her down, but notwithstanding all accidents, she recovered promptly and about a month afterwards gave birth to a living child.

E. L. O'D. had hemorrhage from the nose severely, and was treated similarly, except that I used ergot as the astringent instead of Monsell's solution, applying it to the mucous membrane of the nose by means of an uterine applicator, and then tamponing with cotton saturated with it. It was also given internally in ʒj. doses. Ice was held in the mouth in addition, and cold compresses placed over the nose. I had a short time previously read of the beneficial action of ergot, applied locally to bleeding surfaces, and this case would seem to corroborate it.

J. T., Jr., was treated by tamponing nose with cotton saturated with tr. ferri chlor., which succeeded completely, but I would prefer some other astringent if it could be procured easily, as the excess of muriatic acid renders the preparation a mild caustic.

L. T., a middle-aged woman, with valvular disease of the heart, had profuse epistaxis. I readily arrested it by pushing cotton saturated with Monsell's solution through the nose by means of a female catheter and groove director. The bleeding was from the right nostril, and ran back into the mouth and throat, at first sight appearing to be a severe hemorrhage from the lungs. Two days afterwards I removed the cotton with forceps. The next morning she had a return of the epistaxis, and again lost a large amount of blood; the blood coming from both nostrils. She also swallowed quite a large quantity, and vomited it afterwards. I filled both nostrils from anterior nares to throat with cotton saturated with liq. ferri persulph., and then ordered fl. ext. ergot and fl. ext. hamamelis, fifteen drops of each every hour. I also injected hypodermically several syringefuls of the mixture into her arm in hopes of causing a speedy diminution in the size of the bleeding vessels. There was no further loss of blood, but a cellulitis of the arm, was set up, which caused much trouble, and I would not use the combination again hypodermically, if I could obtain ergot by itself. I allowed the cotton to remain until there was some suppuration, and then removed it, and washed the nose out with water, and injected an astringent lotion.

Dr. M. M. Griffith mentions in *Medical Brief*, July, 1876, having arrested epistaxis with "a piece of fat bacon three or four inches long, cut to fit the nostrils and pushed in tight and far enough to reach the throat. In obstinate cases the bacon may be rolled in (powder) ferri persulph.; the blood may pass down the throat for a short time, but is soon corrected."

*Hemoptysis.*—C. S., a musician, had been ill for a year with either tubercular or syphilitic disease of the lungs. He had been under the treatment of several physicians before I was called to see him. I found him extremely prostrated from severe and protracted hemoptysis. He did not bleed continuously, but would have sudden gushes of blood, and would lose a large amount at a time, then coagulation would occur and the hemorrhage be arrested for twelve or more hours. These attacks usually occurred about 6 o'clock in the evening. I gave him fluid extract of ergot freely by the mouth, as well as gallic acid and other remedies, without effect. I then injected the ergot hypodermically to arrest hemorrhage, which could not be stopped by the same remedy given by the mouth.

*Hemorrhage following Extraction of a Tooth.*—G. H. W. applied to me in September, 1879, for relief from neuralgia of face and head. I examined his mouth, and finding his teeth to be very carious, I directed him to have some of them extracted. He went to a dentist, who extracted the fangs of an upper molar tooth, and found it necessary to break out a piece of the alveolar process. Some hours later he again visited me; he was bleeding freely from a small artery at the apex of the alveolus. I filled the cavity with a cotton compress saturated with Monsell's solution and apparently arrested the hemorrhage, but he returned the next day with his cheek distended with a clot from the iron, but the bleeding was not controlled. I again failed to arrest it with Monsell's solution, and tried nitrate of silver and nitric acid without success. Seeing I could not succeed by styptics, I trimmed a cork to fit the alveolus, inserted it and told him to press upon it as much as possible with his lower jaw. This compression arrested the hemorrhage immediately and permanently, it caused no pain, and gave no inconvenience. The cork was securely retained in situ by the adjoining teeth, and was not removed for nearly a week. Had this method failed, I would probably have applied the actual cautery.

*Hemorrhage from Scalp Wounds, Cured by Acupressure.*—J. M. F., whilst wrestling, fell and cut his head upon the curb stone, making a small wound. He was treated by the police surgeon at the time, but continuing to bleed, he called upon his family physician, who applied a compress and bandage. This answered for awhile, but at intervals of about a week he would have severe hemorrhages from the wound. A month afterwards he was visiting near my office, and whilst sitting quietly in the house, the bleeding commenced. I found the wound filled with fungous granulations, and could not seize the artery. After trying stypics and compression in vain, I transfixed the integument around the wound with two long pins, and tied the skin tightly upon them. The bleeding was arrested at once, and did not recur.

C. C., a young negro, was terribly cut with a razor on September 3d, 1879, upon the head, abdomen and nates, and required twenty pins and stitches to close the wounds. From one of the wounds of the scalp he bled profusely, but it stopped spontaneously. Two weeks afterwards I was called in the middle of the night to arrest hemorrhage from one of the wounds on the head. I found him lying upon the floor in a pool of blood, in a room dimly lighted. I was not able to ligate the artery, therefore transfixed the wound and brought the edges together firmly, and tied the skin upon the pins. This controlled the bleeding, and in a few days the wound healed.

*Wound of Temporal Artery—Compress and Styptic.*—R. F. severed his temporal artery, and was treated by a physician, who applied a bandage and compress, which would arrest the bleeding temporarily, but only for a short time. The bandages were re-applied repeatedly. I was called in the night, and as the wound was fungous, I was unable to put a ligature upon the artery, so I filled the wound with cotton, saturated with Monsell's solution, which made a firm coagulum, and cured him.—*Maryland Medical Journal.*

